# PIAS3 suppresses damage in an Alzheimer’s disease cell model by inducing the STAT3-associated STAT3/Nestin/Nrf2/HO-1 pathway

**DOI:** 10.1186/s10020-021-00410-3

**Published:** 2021-11-27

**Authors:** Chen Li, Ruili Wang, Youyou Zhang, Chunting Hu, Qiaoya Ma

**Affiliations:** Department of Geriatrics Neurology, the Second Affiliated Hospital of Xi’an Jiaotong University, No. 157 Xiwu Road, Xi’an, 710004 Shaanxi People’s Republic of China

**Keywords:** Alzheimer’s disease, Aβ, PIAS3, STAT3, Oxidative, Apoptosis

## Abstract

**Background:**

Alzheimer’s disease (AD), the most common form of dementia, is caused by the degeneration of the central nervous system (CNS). A previous study reported that signal transducer and activator of transcription 3 (STAT3) is activated during AD development; nonetheless, the related mechanism remains unknown. Thus, this study used a cell model to explore whether and how the protein inhibitor of activated STAT3 (PIAS3) is involved in AD development.

**Methods:**

Cerebrospinal fluid (CSF) specimens of 30 patients with AD and 10 normal participants were included in this study. SH-SY5Y cells were used to constructed AD model. Relevant indices were then detected and analyzed.

**Results:**

The results showed that compared with the control group, PIAS3 expression was substantially decreased in patients with AD and amyloid beta (Aβ)-treated SH-SY5Y cells. PIAS3 overexpression was able to reverse the detrimental effects of Aβ treatment on cell survival and growth. Further, it could also ameliorate apoptosis and oxidative stress in Aβ-treated SH-SY5Y cells. Additionally, PIAS3 was shown to reduce the activated form of STAT3 and increase the activity of the downstream Nestin/nuclear factor erythroid 2-related factor/heme oxygenase-1 pathway.

**Conclusions:**

STAT3 reactivation by colivelin treatment negated the influence of PIAS3 on the survival, growth, apoptosis, and oxidative stress of Aβ-treated SH-SY5Y cells.

## Background

Alzheimer’s disease (AD) is characterized clinically by learning incapability and memory loss and pathologically by neurofibrillary tangles, senile plaques, and numerous neuron deficiencies in the cerebral cortex (Mattson 2004). Single-gene mutations on presenilin 1 (*PSEN1*), presenilin 2 (*PSEN2*), and amyloid precursor protein (*APP*) have been identified in cases of autosome-dominant early-onset genetic AD (Karch et al. 2014; Chiba et al. 2008). Amyloid beta (Aβ), derived from APP, is the major component of senile plaques and has long been related to AD occurrence; increased levels of toxic and longer Aβ forms have been consistently observed in many AD models on the basis of familial AD genes (Citron et al. 1997; Bi et al. 2019). Studies have reported that Aβ could result in synaptic spine atrophy, reduce synaptic transmission strength (Selkoe 2008), and damage long-term potentiation induction (Knobloch et al. 2007; Li et al. 2011). Hence, establishing a reliable AD cell model is beneficial for further investigating AD occurrence and treatment. Since Aβ could imitate AD damage, it is commonly utilized to obtain an AD cell model (Villemagne and Masters 2014). Although forced Aβ increase can result in neurotoxicity causing cognitive impairment, the intracellular processes in neurons at high Aβ concentrations remain unclear.

Aberrant signal transducer and activator of transcription 3 (STAT3) signal activation is also relevant to neurodegenerative diseases, including AD and Huntington disease (Guillemaud et al. 2020). Neuropathological analyses have indicated an increase in STAT3 Tyr705 phosphorylation in the hippocampi of patients with AD (Guillemaud et al. 2020; Wan et al. 2010) and marked upregulation of phosphor-STAT3 in APP/PS1 transgenic mice (Wan et al. 2010). This STAT3 activation might induce astrocyte reactivity (Guillemaud et al. 2020), which might decrease ameliorated memory loss (Ceyzériat et al. 2018; Reichenbach 2019). Neuroinflammation elevates proinflammatory cytokine generation and microglial cell overactivation (Heneka et al. 2015). STAT3 could tamper with AD neuroinflammation and beta-site APP cleaving enzyme 1 (BACE1) levels. BACE1 is also modulated at the transcription level, particularly via the activation of STAT3 (Carret-Rebillat et al. 2015; Liu et al. 2013; Wen et al. 2008). STAT3 phosphorylation plays a role in the release of cytokines relevant to AD neuroinflammation, such as tumor necrosis factor (TNF)-α and interleukin (IL)-1β (Samavati et al. 2009).

STAT3 is temporarily activated and inactivated by a series of signal proteins, including protein inhibitor of activated STAT (PIAS) and suppressor of cytokine signaling inhibitors, and Src homology 2 domain-containing protein tyrosine phosphatase (SHP)1 and SHP2 cascades (Saydmohammed et al. 2010). After stimulation by cytokines, PIAS1 and PIAS3 bind to activated STAT1 and STAT3, respectively, and shield them to combine with DNA (Shuai and Liu 2005). Elevated PIAS3 expression has also been observed in many cancers, including cervical (Qu et al. 2019), prostate (Tseng et al. 2020), osteosarcoma (Wang et al. 2017), lung, breast, and brain tumors (Wang and Banerjee 2004); it has also been reported to be relevant to the apoptosis of prostate cancer cells (Wible et al. 2002) and growth inhibition in human lung cancer cells (Ogata, et al. 2006). However, the detailed mechanism of the PIAS3–STAT3 interaction during AD development remains ambiguous.

Furthermore, the role of PIAS3 in the pathogenesis and progression of AD remains unclear. Thus, the present study was aimed at exploring and clarifying the role of PIAS3 on cell survival, growth, oxidative stress, and apoptosis in AD.

## Material and methods

### Patients and ethics

This pilot study was conducted with tissues of patients with AD and normal participants in the Gerontology Department, the Second Affiliated Hospital of Xi’an Jiaotong University (Xi’an City, China), from December 2016 to November 2020. This research included 30 patients with AD (aged 62–81 years). All cerebrospinal fluid (CSF) specimens were harvested at 8:00–9:00 a.m. (Magnin et al. 2017). Normal participants (n = 10, aged 21–35 years) were confirmed not to display any symptoms of nervous system diseases. Exclusion criteria for both patient and control groups included the presence of other diagnosed neurological syndromes. AD patients were diagnosed were diagnosed following the National Institute of Aging and Alzheimer’s Association criteria (McKhann et al. 2011). Cognition was evaluated through the MiniMental State Examination (MMSE) (Cockrell and Folstein 2002), the verbal phonemic fluency (Henry et al. 2004), the Brief Cognitive Battery (Nitrini et al. 2004), and the Frontal Assessment Battery (FAB) (Dubois et al. 2000). These procedures, as well as the MRI, were used for AD patients’ diagnosis. The study was approved by the Institutional Review Board (the Second Affiliated Hospital of Xi’an Jiaotong University) and all participants provided informed consent.

### AD model

SH-SY5Y cells were cultured in complete Dulbecco’s Modified Eagle Medium solution containing fetal bovine serum (10%, Gibco, Rockville, MD, USA), streptomycin (0.1 mg/mL), and penicillin (100 U/mL) and incubated under 5% CO_2_ and saturated humidity. After the cells went through a logarithmic growth phase, Aβ (25 μL, 20 μM) and 0.1% dimethyl sulfoxide (DMSO) were supplemented to the AD model and control groups, respectively. Subsequently, the cells were cultured for another 2 d.

### Transfection and treatment of cells

The cells were seeded into 12-well plates and transfected according to the instructions of Lipofectamine 2000 (Thermo Fisher Scientific, Waltham, MA, USA). The cells were transfected with pcDNA3-PIAS3 and pcDNA3-Empty (Genescript, Shanghai, China). Subsequently, 6 h later, the medium was changed. For STAT3 reactivation, colivelin at a final concentration of 0.5 μM was supplemented to the cells for 12 h.

### Cell survival and colony formation assay

At 24 h after transfection, cells were harvested and inoculated into 96-well plates (1 × 10^4^/mL). The Cell Counting Kit (CCK)-8 assay (Abcam, Cambridge, UK) was used to measure cell viability. In brief, 10 μL of CCK-8 solution was added to cells, which were then incubated at 37 °C for 120 min in the dark. The optical density at 450 nm (OD_450nm_) (i.e., absorbance) was determined using a microplate reader. For the colony formation assay (CFA), cells were cultured for 7 d and subsequently fixed with 4% formaldehyde for 20 min and stained with 1.0% crystal violet.

### Reactive oxygen species measurement

The generation of reactive oxygen species (ROS) was measured using 2'-7'-dichlorofluorescin diacetate. After transfection and treatment, cells were further incubated for 0.5 h at 37 °C in the dark. The fluorescence intensity (Ex 488/Em 525 nm) was measured using a fluorescence microscope (Tokyo, Japan).

### Western blotting

Cells were lysed in radio-immunoprecipitation assay buffer (pH 8.0) and protease inhibitor cocktail (Roche Applied Science). Protein concentration was measured using a bicinchoninic acid (BCA) kit. Protein samples was subsequently subjected to sodium dodecyl sulfate–polyacrylamide gel electrophoresis and transferred to polyvinylidene fluoride membranes (Millipore, MA, USA). After blocking and then incubating the membranes with primary antibodies at 4 °C overnight, the membranes were washed with Tris-buffered saline with Tween 20 (TBST). After incubation with secondary antibodies at room temperature for 1 h, the protein bands were detected. After rinsing few times with TBST, bands were visualized through the relevant kit.

### RNA isolation and quantitative polymerase chain reaction

Total RNA was extracted using TRIzol (Invitrogen, USA) from CSF samples (200–250 mg) and SH-SY5Y cells (4 × 10^6^ cells), and its concentration was measured. cDNA was obtained through reverse transcription via Oligo (dT) 20 primer (Invitrogen, USA) and MMLV First-Strand Kit. mRNAs were detected via quantitative polymerase chain reaction (qPCR) using SYBR Select Master Mix (Invitrogen, USA), and all experiments were performed under the relevant guidance. The reaction conditions were as follows: first denaturation (95 °C, 10 min), 40 denaturation cycles (95 °C, 15 s), and extension (60 °C, 40 s). The expressions of target mRNAs were measured through the 2-ΔΔCT method with glyceraldehyde 3-phosphate dehydrogenase (GAPDH) mRNA as the internal control. The primers sequences were showed in Table 1. All experiments were conducted with three simultaneous replicates.

**Table 1:** The sequence of primers

Primer	Sequences
PIAS3 F	5’-TGT CAC CAT GAA ACC ATT GC-3’
PIAS3 R	5’-AGG TAA AGT GCG CTT CCT CA-3’
Bcl-2 F	5’-CAT TTC CAC GTC AAC AGA ATT G-3’
Bcl-2 R	5’-AGC ACA GGA TTG GAT ATT CCA T-3’
Bax F	5’-AGC TGA GCG AGT GTC TCA AG-3’
Bax R	5’-GTC CAA TGT CCA GCC CAT GA-3’
STAT3 F	5’-CTT TGA GAC CGA GGT GTA TCA CC-3’
STAT3 R	5’-GGT CAG CAT GTT GTA CCA CAG G-3’
Nestin F	5’-CTG CTA CCC TTG AGA CAC CTG-3’
Nestin R	5’-GGG CTC TGA TCT CTG CAT CTA C-3’
Nrf2 F	5’-ACA CGG TCC ACA GCT CAT C-3’
Nrf2 R	5’-TGT CAA TCA AAT CCA TGT CCT G-3’
HO-1 F	5’-AAC TTT CAG AAG GGC CAG GT-3’
HO-1 R	5’-CTG GGC TCT CCT TGT TGC-3’
GAPDH F	5’-CTG ACT TCA ACA GCG ACA CC-3’
GAPDH R	5’-TGC TGT AGC CAA ATT CGT TGT-3’

### Cell apoptosis

Flow cytometry (FC) was performed to evaluate cell apoptosis. After suspending the cells in binding buffer (20 µL), the cells were exposed to annexin V-FITC/PI (10 µL/5 µL). The apoptosis rate was determined using a flow cytometer.

### Data analysis

All results are displayed as means ± standard deviation. One-way analysis of variance and t test were used to determine differences among multiple groups and differences between two groups, respectively. P-values of < 0.05 were considered statistically significant.

## Results

### Different PIAS3 expression in patients with AD and model

**Fig. 1:**
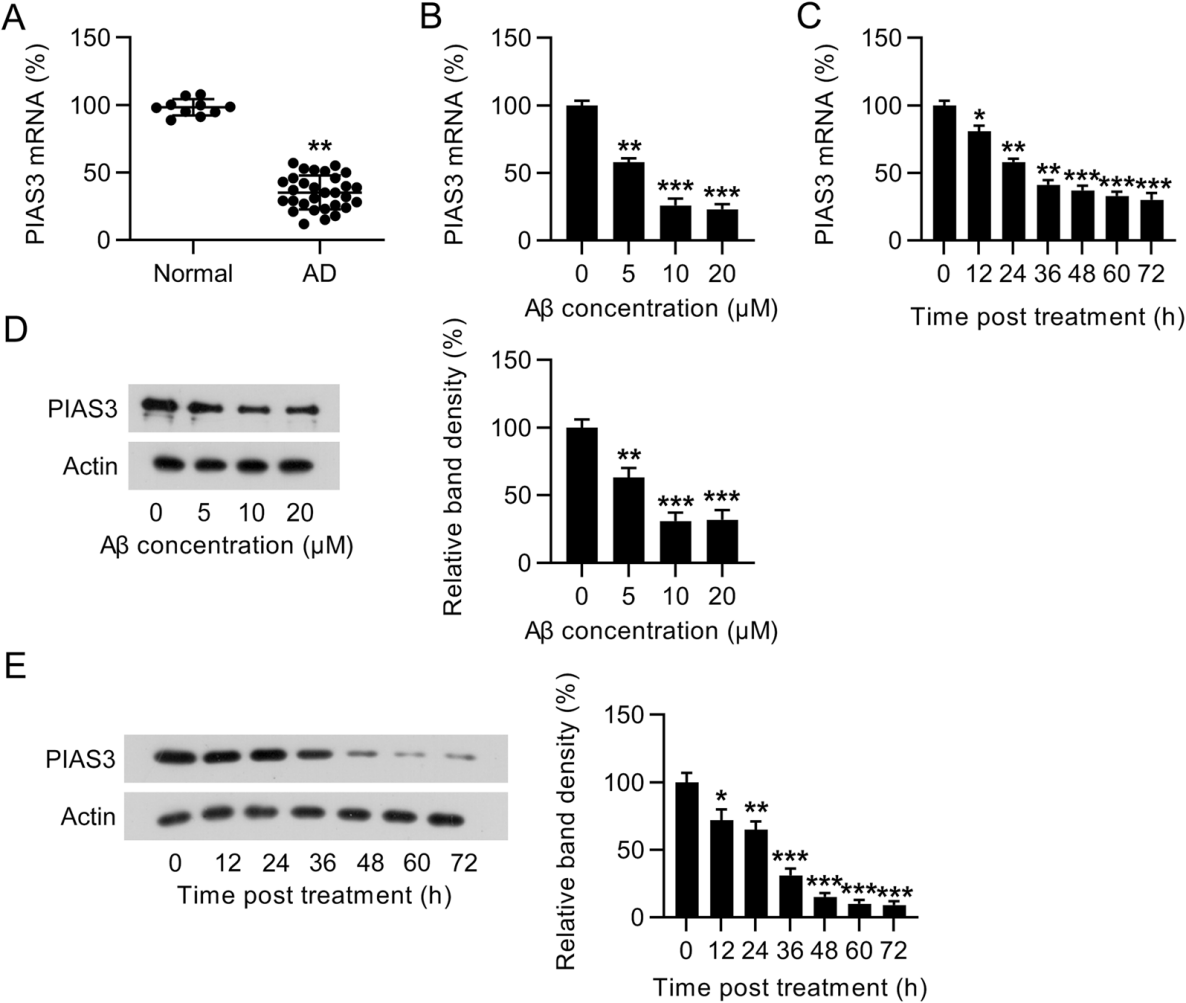
PIAS3 expression in CSF tissues of patients with AD and AD model. **A** qPCR analysis revealed PIAS3 expression in CSF tissues of patients with AD (n = 30) and normal participants (n = 10). **B**–**E** SH-SY5Y cells were treated with Aβ (0, 5, 10, 20 μM in 0.1% DMSO) for 0, 12, 24, 36, 48, 60, and 72 h. qPCR and WB revealed the expression levels of PIAS3 in SH-SY5Y cells at the mRNA and protein levels. Data represent mean ± SEM. *p < 0.05, **p < 0.01, ***p < 0.001. *PIAS3* protein inhibitor of activated STAT3, *CSF* cerebrospinal fluid, *qPCR* quantitative polymerase chain reaction, *AD* Alzheimer’s disease, *Aβ* amyloid beta, *DMSO* dimethyl sulfoxide, *WB* western blotting

CSF samples were obtained from patients with AD and utilized for PIAS3 expression analysis. The PIAS3 mRNA expression in CSF tissues of patients with AD was markedly decreased relative to that in CSF tissues of healthy volunteers (Fig. 1A). SH-SY5Y cells treated with Aβ were used to establish the AD cell model (Liu et al. 2018). Reduced expression of PIAS3 mRNA in AD cell model was observed in a dose- and time-dependent manner, at both mRNA and protein levels (Fig. 1B–E). The results demonstrated that PIAS3 expression reached bottom at 36–48 h post Aβ treatment at the concentration of 10 μM. These findings indicate that PIAS3 expression in AD tissues and cell model is reduced.

### PIAS3 overexpression restored cell viability and alleviated apoptosis and inflammation of Aβ-treated SH-SY5Y cells

**Fig. 2:**
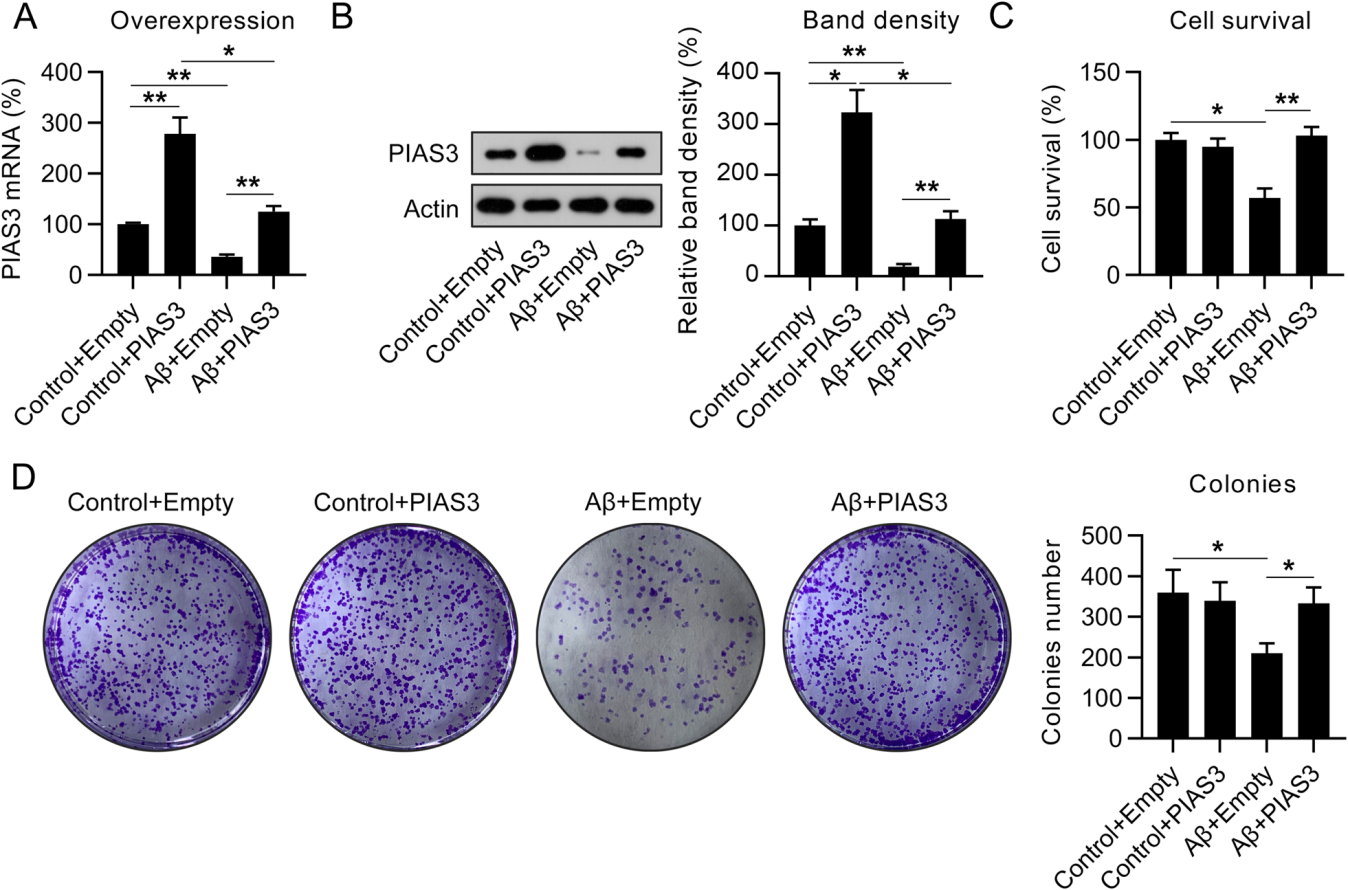
Effects of PIAS3 overexpression on the survival of Aβ-treated SH-SY5Y cells. Cells were subjected to transfection with a PIAS3 overexpression vector or an empty vector for 1 d, and subsequently subjected to Aβ treatment (20 μM in 0.1% DMSO) for an additional 1.5 d. **A**, **B** qPCR and WB were performed to determine PIAS3 expression. **C** The CCK-8 assay revealed that effects of PIAS3 on cell survival 2 d after treatment. **D** CFA showed the influence of PIAS3 upregulation on cell growth. Data represent mean ± SEM. *p < 0.05, **p < 0.01. *PIAS3* protein inhibitor of activated STAT3; *qPCR* quantitative polymerase chain reaction, *Aβ* amyloid beta, *WB* western blotting, *CCK-8* Cell Counting Kit-8, *DMSO* dimethyl sulfoxide, *CFA* colony formation assay

Cells were transfected with a PIAS3 overexpression vector or an empty vector for 36 h. The results of qPCR and western blotting (WB) indicated that the mRNA and protein levels of PIAS3 were dramatically elevated owing to the transfection of the PIAS3 overexpression vector (Fig. 2A, B).

Regarding cell viability, the CCK-8 assay revealed that Aβ treatment considerably decreased the survival of SH-SY5Y cells, whereas PIAS3 overexpression dramatically restored the viability of Aβ-treated SH-SY5Y cells, compared with nontreated cells (Fig. 2C). In addition, CFA revealed that the elevation of PIAS3 expression visibly increased the number of colonies observed for Aβ-impaired SH-SY5Y cells (Fig. 2D). Further, PIAS3 overexpression did not impact nontreated cells, suggesting that the protective role of PIAS3 was specific to Aβ-treated cells.

**Fig. 3:**
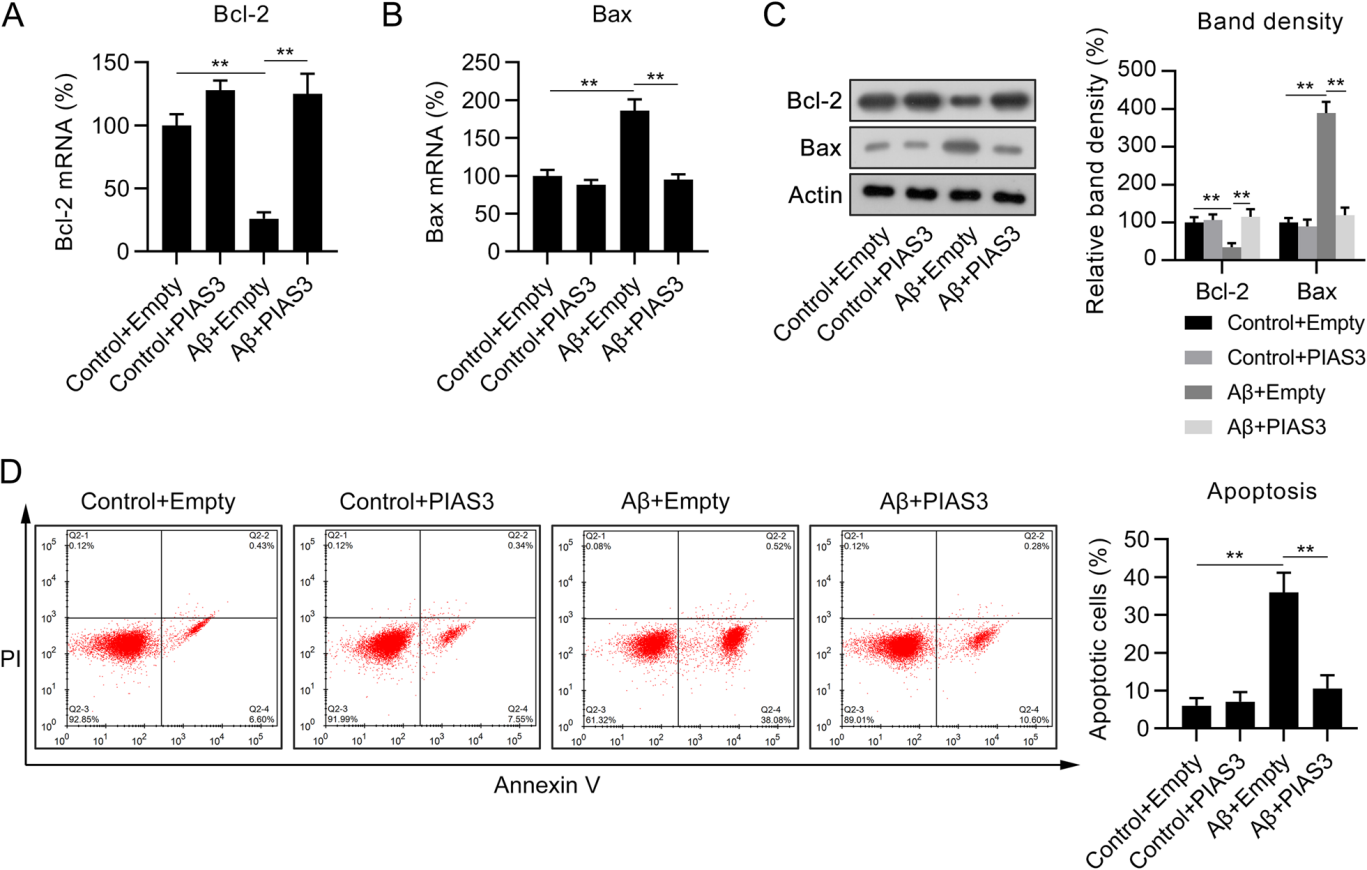
Effects of PIAS3 overexpression on the apoptosis of Aβ-treated SH-SY5Y cells. Cells were subjected to transfection with a PIAS3 overexpression vector or an empty vector for 1 d, and subsequently subjected to Aβ treatment (20 μM in 0.1% DMSO) for an additional 1.5 d. **A**–**C** qPCR and WB were used for determining Bcl-2 and Bax mRNA and protein expression levels. **D** The number of apoptotic cells was determined using annexin V/PI FC. Data represent mean ± SEM. **p < 0.01. *PIAS3* protein inhibitor of activated STAT3, *qPCR* quantitative polymerase chain reaction, *Aβ* amyloid beta, *WB* western blotting, *Bcl-2* B-cell lymphoma 2, *Bax* bcl-2-like protein 4, *DMSO* dimethyl sulfoxide, *FC* flow cytometry

Apoptosis plays a major role in Aβ-induced attenuation of cell viability; therefore, the relevance of PIAS3 to the apoptosis of Aβ-treated SH-SY5Y cells was assessed. qPCR and WB were performed to determine the expressions of B-cell lymphoma 2 (Bcl-2) and bcl-2-like protein 4 (Bax) in cells. As a result, Aβ was found to induced a decrease in Bcl-2 and an increase in Bax at the mRNA and protein levels. PIAS3 overexpression dramatically restored Bcl-2 expression but decreased Bax levels (Fig. 3A–C). Furthermore, annexin V/PI FC revealed that Aβ treatment induced apoptosis in SH-SY5Y cells, compared with nontreated cells. In cells with PIAS3 overexpression, the proapoptotic effect of Aβ treatment was partially abolished (Fig. 3D), indicating that PIAS3 suppressed apoptosis of Aβ-treated SH-SY5Y cells.

**Fig. 4:**
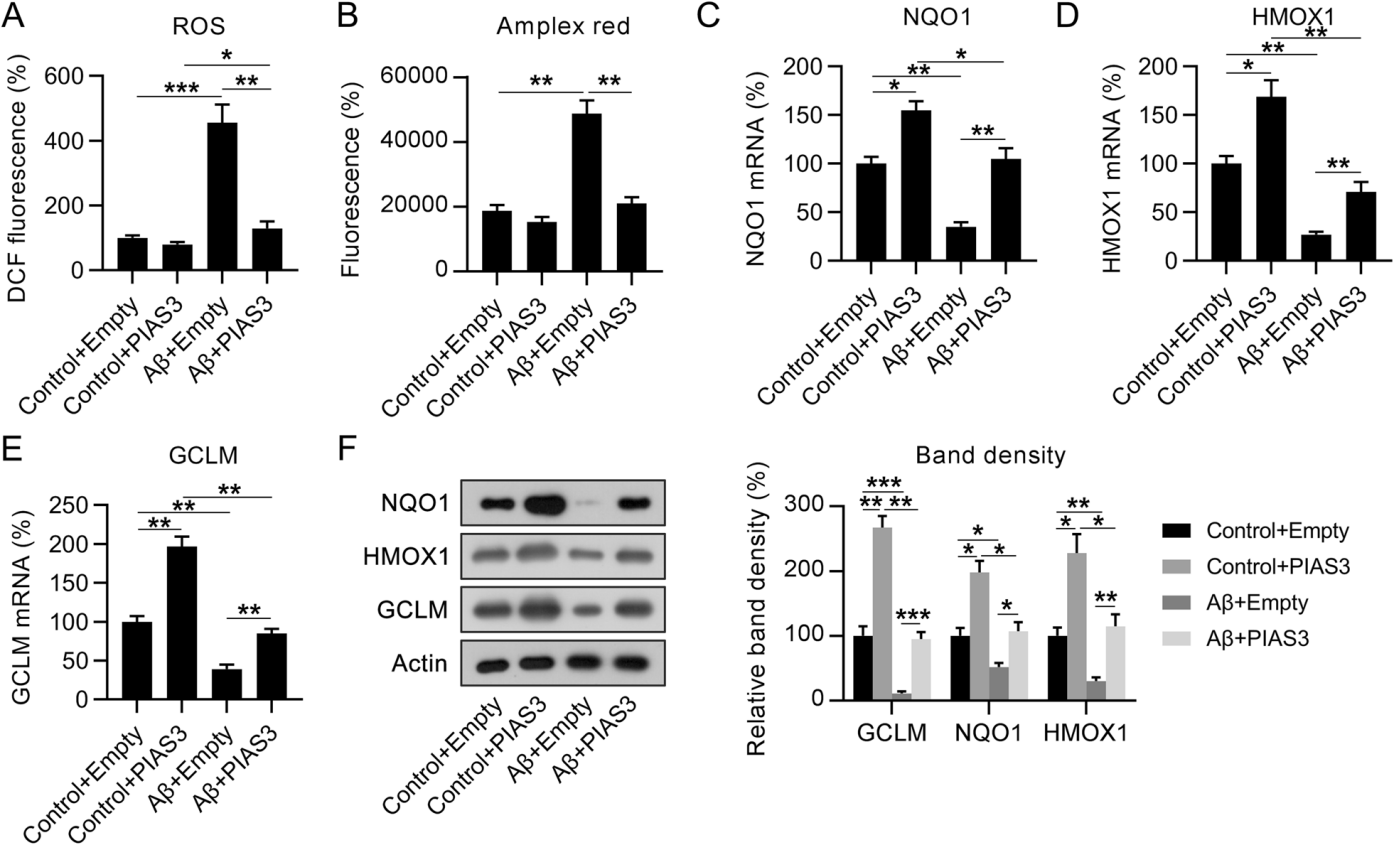
Effects of PIAS3 overexpression on oxidative stress in Aβ-treated SH-SY5Y cells. Cells were subjected to transfection with a PIAS3 overexpression vector or an empty vector for 2 d, and subsequently subjected to Aβ treatment (20 μM in 0.1% DMSO) for an additional 1.5 d. **A**, **B** ROS generation was determined by DCF-fluorescence and Amplex red stain at 24 h post transfection. **C**–**F** qPCR and WB were performed for determining the levels of NQO1, HMOX1, and GCLM in SH-SY5Y cells. Data represent mean ± SEM. *p < 0.05, **p < 0.01, ***p < 0.001. *PIAS3* protein inhibitor of activated STAT3, *qPCR* quantitative polymerase chain reaction, *Aβ* amyloid beta, *WB* western blotting, *ROS* reactive oxygen species, *DMSO* dimethyl sulfoxide

Aβ induces neuronal oxidative stress during AD development (Felice et al. 2007), and oxidative stress can cause apoptosis resulting in neuronal injury (Poh Loh et al. 2006). We first determined whether PIAS3 was involved in Aβ-induced oxidative stress. Regarding ROS production, the Aβ treatment resulted in obvious and robust ROS generation in SH-SY5Y cells, which was partially downregulated after PIAS3 overexpression (Fig. 4A, B). In contrast, qPCR and WB revealed that Aβ treatment caused significant downregulation of three antioxidant genes, *NQO1*, *HMOX1*, and *GCLM*. However, PIAS3 overexpression upregulated the expression of these genes in both Aβ- and nontreated cells (Fig. 4C–F), suggesting that PIAS3 can reduce Aβ-induced oxidative stress.

### PIAS3 activated the STAT3/Nestin/Nrf2/HO-1 pathway in Aβ-treated SH-SY5Y cells

**Fig. 5:**
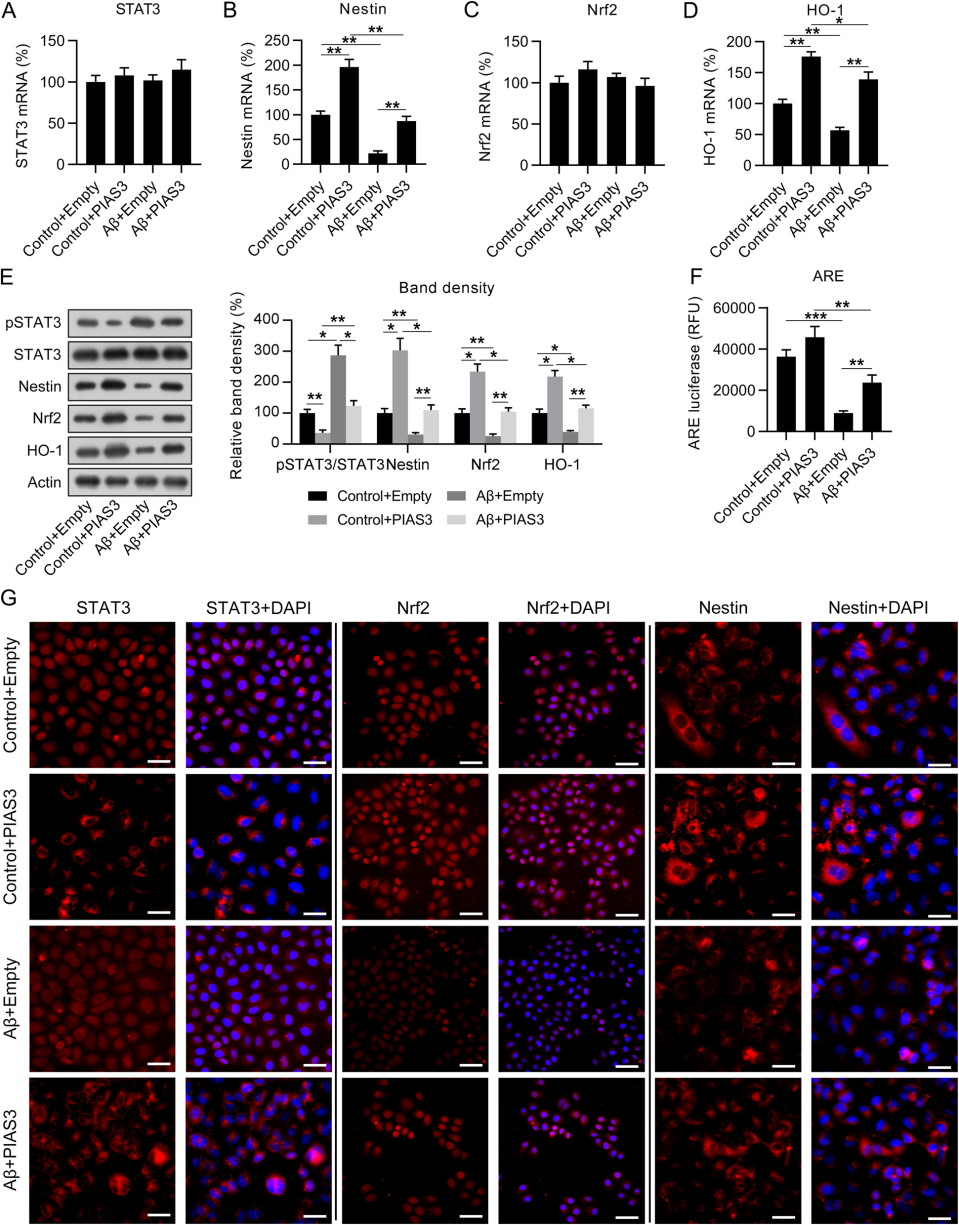
Effects of PIAS3 overexpression on STAT3 activation and Nestin/Nrf2/HO-1 pathway in Aβ-treated SH-SY5Y cells. **A**–**D** qPCR was used to determine the mRNA level expressions of STAT3, Nestin, Nrf2, and HO-1. **E** WB was utilized for determining the protein level expressions of STAT3, Nestin, Nrf2, and HO-1, and STAT3 phosphorylation. **F** ARE luciferase activity was also determined. **G** Fluorescence microscopy was performed to detect the expression and location of STAT3, Nrf2, and Nestin in cells. Scale bar, 50 μm. *P < 0.05, **P < 0.01 vs. indicated groups. Data represent mean ± SEM. *p < 0.05, **p < 0.01, ***p < 0.001. *PIAS3* protein inhibitor of activated STAT3, *STAT3* signal transducer and activator of transcription 3, *Nrf2* nuclear factor erythroid 2-related factor, *HO-1* heme oxygenase-1, *qPCR* quantitative polymerase chain reaction, *Aβ* amyloid beta, *WB* western blotting, *ARE* antioxidant responsive element

Nrf2 is a well-known modulator of ROS homeostasis (Ma 2013), and Nestin and HO-1 are its upstream (Wang et al. 2019) and downstream effectors (Jeong et al. 2019), respectively. Therefore, we hypothesized that PIAS/STAT3 dysregulation-associated ROS generation might be modulated via the Nestin/Nrf2/HO-1 pathway during AD development. We determined the expressions of STAT3, Nestin, Nrf2, and HO-1 mRNAs in Aβ-treated SH-SY5Y cells. qPCR revealed that STAT3 and Nrf2 mRNA levels were not altered in the cells, regardless of Aβ treatment and PIAS3 overexpression (Fig. 5A, C). Nestin and HO-1 mRNA levels were significantly reduced by Aβ treatment, but this reduction could be reversed by PIAS3 overexpression (Fig. 5B, D). Regarding the protein level expressions of these genes, WB revealed that Aβ treatment induced the phosphorylation of STAT3, whereas PIAS3 upregulation significantly caused a reduction of phosphorylated STAT3. Meanwhile, the protein level of Nestin was consistent with its mRNA level. The protein levels of Nrf2 and HO-1, which operate downstream of Nestin, were found to be reduced after Aβ treatment, but this downregulation was reversed by PIAS3 overexpression (Fig. 5E). Furthermore, the antioxidant responsive element (ARE) is an enhancer element, which can be activated by Nrf2 (Johnson et al. 2008). A luciferase reporter system reflecting ARE activation showed that Aβ treatment caused a decrease in ARE activity; in contrast, PIAS3 overexpression upregulated the ARE luciferase signals (Fig. 5F). Furthermore, fluorescence microscopy was performed to detect the expression and location of STAT3, Nrf2, and Nestin in cells. Cytoplasmic location of STAT3 could be induced by PIAS3 overexpression. Nuclear Nrf2 expression and cytoplasmic Nestin were reduced by Aβ treatment, but restored after PIAS3 overexpression (Fig. 5G) suggesting that the Nestin/Nrf2/HO-1 pathway may be positively correlated with PIAS3 expression in Aβ-treated SH-SY5Y cells through STAT3 phosphorylation.

## Involvement of STAT3 activation in PIAS3-mediated cell viability, oxidative stress, and apoptosis of Aβ-treated SH-SY5Y cells

**Fig. 6:**
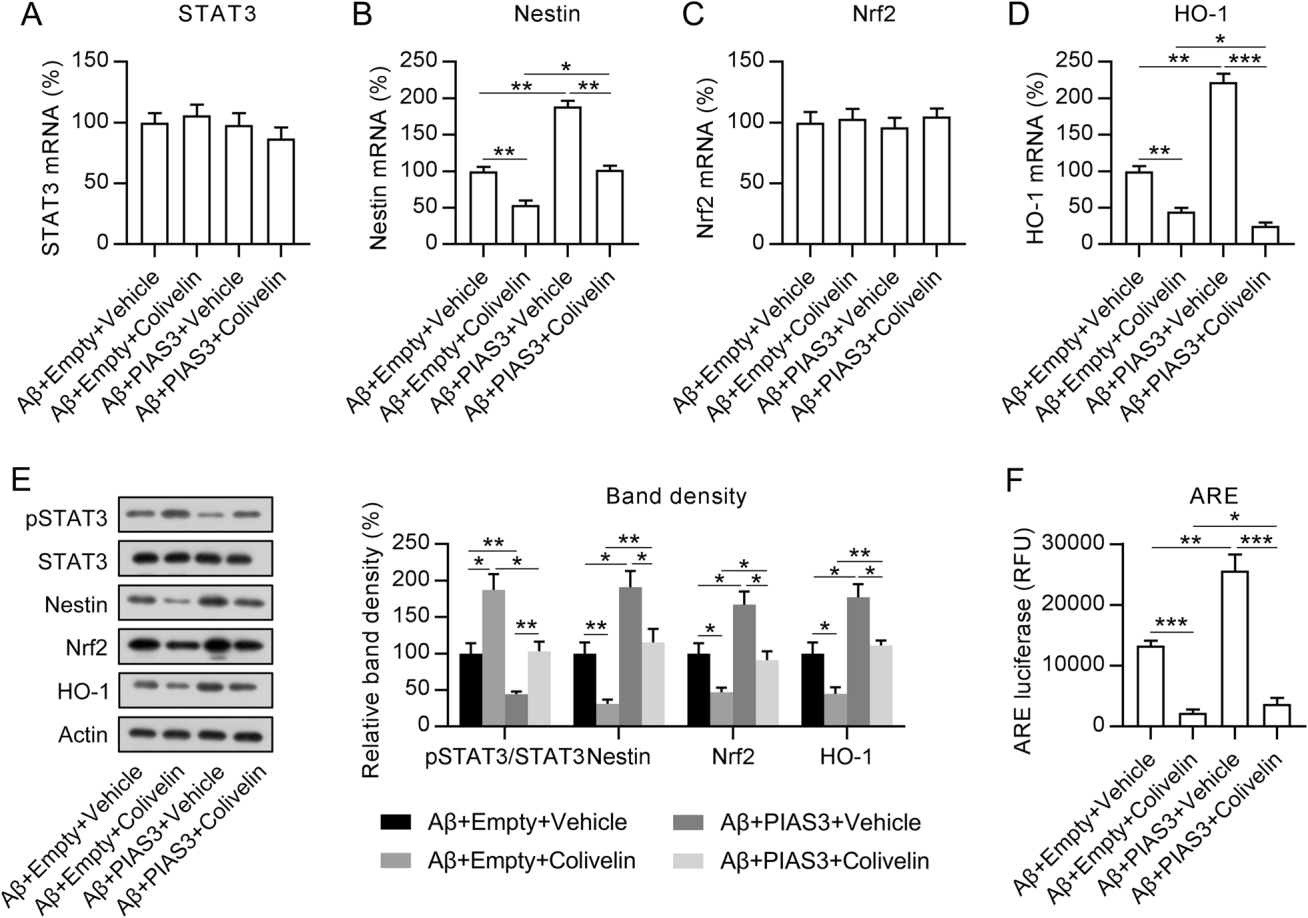
Effects of colivelin treatment on STAT3 activation and Nestin/Nrf2/HO-1 pathway in Aβ-treated SH-SY5Y cells. Cells were subjected to transfection with a PIAS3 overexpression vector or an empty vector for 1 d, and subsequently subjected to Aβ treatment (20 μM in 0.1% DMSO) and colivelin treatment (0.5 μM in 0.1% DMSO) for an additional 1 d. **A**–**D** qPCR was used to determine mRNA level expressions of STAT3, Nestin, Nrf2, and HO-1. **E** WB was utilized for determining protein level expressions of STAT3, Nestin, Nrf2, and HO-1, and STAT3 phosphorylation in SH-SY5Y cells. **F** ARE luciferase activity was also determined. Data represent mean ± SEM. **p < 0.01, ***p < 0.001. PIAS3, protein inhibitor of activated STAT3, *STAT3* signal transducer and activator of transcription 3, *Nrf2* nuclear factor erythroid 2-related factor, *HO-1* heme oxygenase-1, *qPCR* quantitative polymerase chain reaction, *Aβ* amyloid beta, *WB* western blotting, *ARE* antioxidant responsive element, *DMSO* dimethyl sulfoxide

To further investigate the role of STAT3 activation in the viability, oxidative stress, and apoptosis of Aβ-treated SH-SY5Y cells, the cells were first subjected to transfection with a PIAS3 overexpression vector for 1 d and then exposed to STAT3 activator colivelin for an additional 12 h. qPCR and WB data confirmed that the mRNA levels of STAT3 and Nrf2 were not altered by colivelin (Fig. 6A, C) but those of Nestin and HO-1 were significantly decreased (Fig. 6B, D). WB revealed the deactivation of the Nestin/Nrf2/HO-1 pathway, as evidenced by the downregulation of these three proteins (Fig. 6E). In addition, ARE luciferase activity displayed a reduced luminescence signal (Fig. 6F), indicating that STAT3 activation blocked the Nestin/Nrf2/HO-1 pathway.

**Fig. 7:**
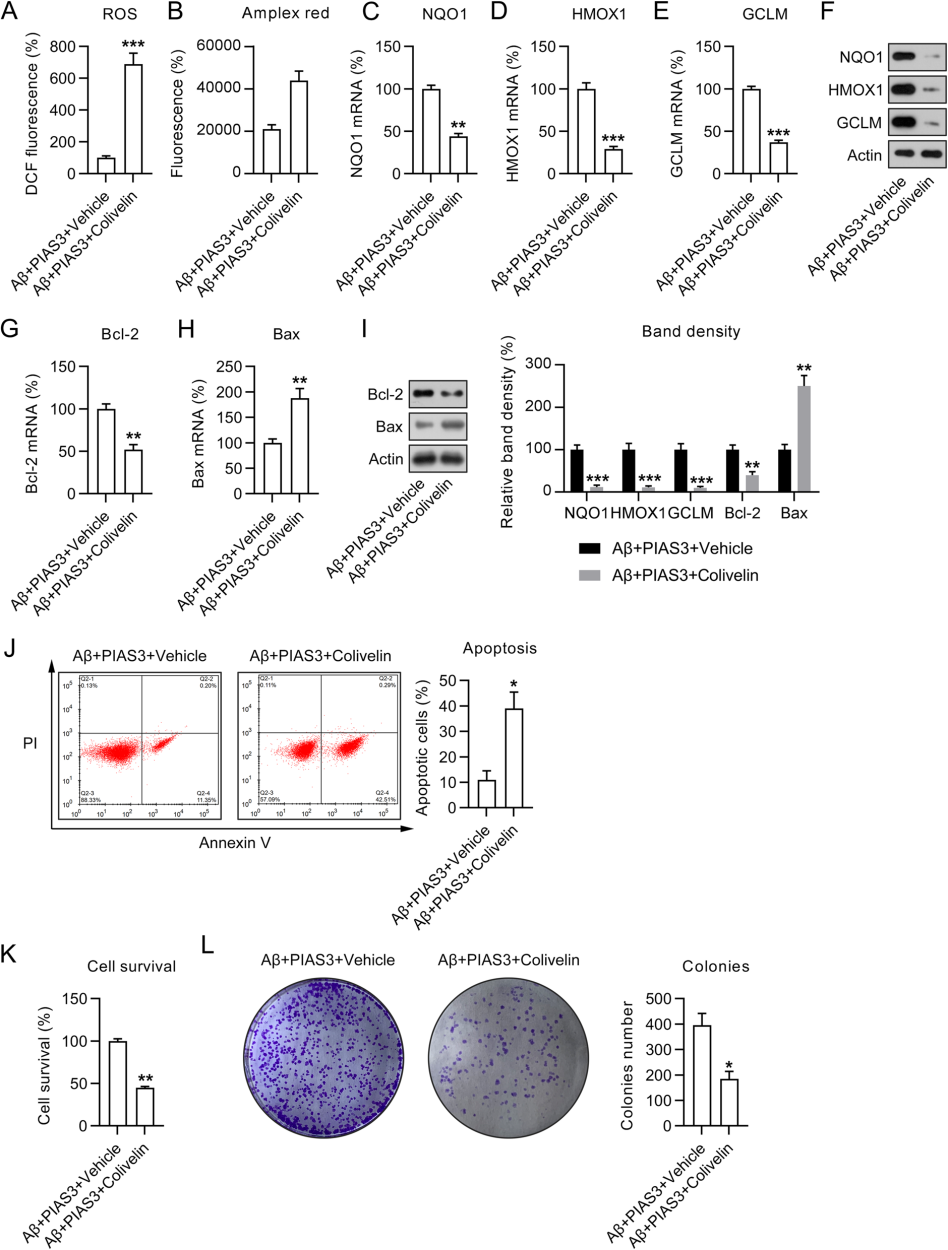
Effects of colivelin treatment on cell survival, oxidative stress, and apoptosis in Aβ-treated and PIAS3-overexpressing SH-SY5Y cells. Cells were subjected to transfection with a PIAS3 overexpression vector or an empty vector for 1 d, and subsequently subjected to Aβ treatment (20 μM in 0.1% DMSO) and colivelin treatment (0.5 μM in 0.1% DMSO) for an additional 1 d. **A**, **B** ROS generation was determined by DCF-fluorescence and Amplex red stain at 24 h post transfection. **C**–**F** qPCR and WB were utilized for determining the expression levels of NQO1, HMOX1, and GCLM in SH-SY5Y cells. **G**–**I** qPCR and WB were used for determining Bcl-2 and Bax mRNA and protein expressions. **J** The number of apoptotic cells was determined using annexin V/PI FC. **K** The CCK-8 assay demonstrated the influence of PIAS3 on cell survival 2 d after treatment. **L** CFA showed the influence of PIAS3 upregulation on cell growth of SH-SY5Y cells. Data represent mean ± SEM. *p < 0.05, **p < 0.01, ***p < 0.001. *PIAS3* protein inhibitor of activated STAT3, *qPCR* quantitative polymerase chain reaction, *Aβ* amyloid beta, *WB* western blotting, *ROS* reactive oxygen species, *Bcl-2* B-cell lymphoma 2, *Bax* bcl-2-like protein 4, *CCK-8* Cell Counting Kit-8, *CFA* colony formation assay, *FC* flow cytometry, *DMSO* dimethyl sulfoxide

As STAT3 activation blocked the Nestin/Nrf2/HO-1 pathway, we next determined whether colivelin could hinder the amelioration of oxidative stress by PIAS3. We found that ROS generation in Aβ-treated and PIAS3-overexpressing cells was significantly upregulated following colivelin treatment (Fig. 7A, B). Meanwhile, the expressions of the three antioxidant genes (*NQO1*, *HMOX1*, and *GCLM*) were decreased by colivelin at both mRNA and protein levels (Fig. 7C–F), suggesting that STAT3 reactivation counteracted PIAS3’s role in the antioxidant response.

Moreover, the results of qPCR and WB showed that colivelin treatment contributed to the obvious decrease of Bcl-2 and increase of Bax in Aβ-treated and PIAS3-overexpressing SH-SY5Y cells, consequently negating the influence of PIAS3 on these factors (Fig. 7G–I). Besides, STAT3 reactivation also upregulated the apoptosis rate, which was inhibited by PIAS3 (Fig. 7J).

Furthermore, the role of STAT3 phosphorylation in cell survival and growth was assessed. The CCK-8 assay indicated that colivelin could significantly attenuate cell viability in colivelin-treated group relative to the vehicle-treated (DMSO-treated) group (Fig. 7K). CFA showed that the colivelin-treated group displayed a decreased cell growth rate relative to the vehicle-treated group (Fig. 7L). Therefore, these findings suggest that STAT3 activation was involved in PIAS3-mediated cell survival, apoptosis, and oxidative stress.

## Discussion

AD is a type of neurodegenerative disease that mainly occurs in older persons. Its main clinical manifestation involves progressive cognitive injury and memory loss (Brookmeyer et al. 2011). The pathological changes in AD include gradual loss of neurons and synapses in the cerebral cortex and hippocampus, which is related to transient memory loss and cognitive impairment. At the molecular level, AD pathologically manifests senile plaques resulting from insoluble Aβ outside cells and neurofibrillary tangles resulting from Tau protein hyperphosphorylation (Marcus and Schachter 2011). Hence, exploring the occurrence of AD is critical for the discovery of novel treatment targets for AD. Many studies have shown that Aβ is an important factor involved in AD occurrence. Aβ is capable of activating microglia and astrocytes resulting in inflammation, gradually leading to synaptic impairment. It changes the balance among neurons and results in oxidative stress, thereby slowly causing dementia (Villemagne and Masters 2014). Thus, we established an AD model using Aβ-treated SH-SY5Y cells. The present data revealed that PIAS3 expression was dramatically decreased in patients with AD. Moreover, PIAS3 overexpression markedly restored cell survival and ameliorated oxidative stress and apoptosis in Aβ-treated cells. PIAS3 upregulation also deactivated STAT3 and activated the Nestin/Nrf2/HO-1 pathway in Aβ-treated SH-SY5Y cells. Reactivation of STAT3 by colivelin treatment resulted in the blockage of Nestin/Nrf2/HO-1 signal transduction and partially abolished the effects of PIAS3 overexpression in Aβ-treated SH-SY5Y cells.

STAT3 can regulate Aβ production and astrocyte proliferation and neurotoxicity as a transcriptional factor (Hashioka et al. 2011). STAT3 deletion in astrocytes downregulates proinflammatory responses, elevates Aβ clearance, and protects APP/PS1 mice from memory loss (Reichenbach, et al. 2019). STAT3 activation in the brains of AD models reveal obvious nuclear staining among nonneuronal cells. STAT3 can also facilitate synaptic plasticity (Nicolas et al. 2012) and neurogenesis (Chen et al. 2013), which are processes mainly implicated in memory and learning (Saxe et al. 2006). Further, although pharmacological STAT3 suppression by AG490 shields memory loss, AG490 per se has been reported to lead to memory injury in controls. In summary, these findings reveal that memory function requires fine modulation of STAT3 phosphorylation levels. Considering the specific role of PIAS3 in the suppression of STAT3 activation, we hypothesized that PIAS3 also participates in different processes during AD development via the downregulation of STAT3 phosphorylation. Here we observed downregulation of PIAS3 in the CSF samples of patients with AD and in the AD cell model. Aβ-induced cell injury, which is highly associated with oxidative stress and apoptosis, can be alleviated by PIAS3 overexpression. Notably, the overexpression of PIAS3 caused dephosphorylation and deactivation of STAT3, which may account for the positive effects of PIAS3 on the restoration of Aβ-treated SH-SY5Y cells.

Nestin is a common marker of multipotent stem cells (Jiang et al. 2014) that, according to extensive reports, is dysregulated by tissue injuries and cancer progression (Tampaki 2014). Previous reports have clarified the underlying molecular mechanisms of Nestin in cancer development. In the process of differentiation of neural precursor cells (NPCs), STAT3 is present and active in the developing mouse CNS and is accompanied by the expression of the neural stem cell marker, Nestin (Foshay and Gallicano 2008), suggesting a positive correlation between these two proteins. Herein, our data demonstrated that corresponding with STAT3 deactivation via PIAS3 overexpression in Aβ-treated SH-SY5Y cells, Nestin was dramatically increased at the mRNA and protein levels. In contrast, colivelin induced the phosphorylation of STAT3 and abated Nestin upregulation. These data suggested a converse correlation between STAT3 phosphorylation and Nestin expression, which is inconsistent with the previous study (Foshay and Gallicano 2008). This inconsistency may be attributed to usage of different cell lines and treatment methods.

An abundance of ROS can result in neuron death and brain function alterations (Anderson and Maes 2014). Therefore, neurodegenerative diseases, such as AD (Aslan and Ozben 2004) and Parkinson’s disease (Jenner 2003), are commonly characterized by upregulated oxidative markers and a lack of enzyme antioxidant systems. Nrf2 activation is one reason for neuroprotection; Nrf2 is a general transcription factor that can regulate oxidative stress response. It can regulate detoxifying, antioxidant, and anti-inflammatory genes by combining with AREs (Gan and Johnson 2014), which are enhancer sequences existing in the regulatory regions of Nrf2 target genes, including HO-1 (Ali et al. 2018). Nrf2 expression has been observed in neurons and glial cells in brains, but it is more often expressed in astrocytes; further, endogenous Nrf2 expression and activation are evidently observed in neurons in the process of neurodegeneration and aging (Liddell 2017). Nrf2 activation can also shield against mitochondrial toxins in the primary neuronal medium (Lee et al. 2003). In lung cancer, Nestin was reportedly capable of protecting Nrf2 from kelch-like ECH-associated protein 1 (Keap1)-modulated degradation and was able to increase the expression levels of antioxidant enzymes. Nestin directly combines with both Keap1 and Nrf2, and increased Nrf2 expression at the protein level has been shown to modulate the oxidative equilibrium in lung cancer (Wang, et al. 2019), suggesting that Nestin is an upstream modulator of Nrf2. The present data showed that Nestin/Nrf2/HO-1 and ARE were activated via PIAS3 overexpression and blocked by colivelin treatment. Signal transduction in this pathway was accompanied by the recovery of Aβ-treated SH-SY5Y cell viability. We hypothesized that Aβ treatment caused robust ROS production in the cells, which subsequently triggered apoptosis and cell damage. PIAS3 overexpression resulted in the dephosphorylation of STAT3, which consequently mediated Nestin expression. A high level of active Nrf2 protein resulting from the activity of Nestin protected the cells against ROS-triggered cell death.

## Conclusion

In summary, this study found that PIAS3 increased cell survival and antioxidant response and prevented apoptosis of Aβ-treated SH-SY5Y cells via regulating STAT3/Nestin/Nrf2/HO-1 signal pathway.Based on these data, it may be hypothesized that PIAS3/STAT3 and Nestin/Nrf2/HO-1 crosstalk occurs in patients with AD. Nonetheless, further studies, in particular animal-based experiments, are warranted to clarify the roles of PIAS3 in AD animal models.

## Data Availability

The data used to support the findings of this study are available from the corresponding author upon request.

## Abbreviations

AD: Alzheimer’s disease
CNS: Central nervous system
STAT3: Signal transducer and activator of transcription 3
PIAS3: Protein inhibitor of activated STAT3
Aβ: Amyloid beta
*PSEN1*: Presenilin 1
*PSEN2*: Presenilin 2
*APP*: Amyloid precursor protein
BACE1: Beta-site APP cleaving enzyme 1
TNF: Tumor necrosis factor
IL: Interleukin
SHP: Src homology 2 domain-containing protein tyrosine phosphatase
CSF: Cerebrospinal fluid
CCK: Cell Counting Kit
CFA: Colony formation assay
ROS: Reactive oxygen species
BCA: Bicinchoninic acid
TBST: Tris-buffered saline with Tween 20
qPCR: Quantitative polymerase chain reaction
FC: Flow cytometry
WB: Western blotting
Bcl-2: B-cell lymphoma 2
Bax: Bcl-2-like protein 4
